# Seizure Prediction in EEG Signals Using STFT and Domain Adaptation

**DOI:** 10.3389/fnins.2021.825434

**Published:** 2022-01-18

**Authors:** Peizhen Peng, Yang Song, Lu Yang, Haikun Wei

**Affiliations:** ^1^Key Laboratory of Measurement and Control of Control Science and Engineering (CSE), Ministry of Education, School of Automation, Southeast University, Nanjing, China; ^2^State Grid Nanjing Power Supply Company, Nanjing, China; ^3^Epilepsy Center, the Affiliated Brain Hospital of Nanjing Medical University, Nanjing, China

**Keywords:** seizure prediction, feature extraction, neuropsychiatric disorders, domain adaptation, STFT, EEG

## Abstract

Epileptic seizure prediction is one of the most used therapeutic adjuvant strategies for drug-resistant epilepsy. Conventional approaches commonly collect training and testing samples from the same patient due to inter-individual variability. However, the challenging problem of domain shift between various subjects remains unsolved, resulting in a low conversion rate to the clinic. In this work, a domain adaptation (DA)-based model is proposed to circumvent this issue. The short-time Fourier transform (STFT) is employed to extract the time-frequency features from raw EEG data, and an autoencoder is developed to map these features into high-dimensional space. By minimizing the inter-domain distance in the embedding space, this model learns the domain-invariant information, such that the generalization ability is improved by distribution alignment. Besides, to increase the feasibility of its application, this work mimics the data distribution under the clinical sampling situation and tests the model under this condition, which is the first study that adopts the assessment strategy. Experimental results on both intracranial and scalp EEG databases demonstrate that this method can minimize the domain gap effectively compared with previous approaches.

## 1. Introduction

### 1.1. Epilepsy Background

Epilepsy is a brain disorder characterized by the transient occurrence of unexpected seizures, which stems from excessive, or hypersynchronous neuronal activities (Fisher et al., 2005). It affects approximately 1.0% of the world's population (Banerjee et al., 2009), and around half of them experience severe seizures. Besides, although the anti-epileptic drug (AED) administration is applied to patients, about 30% of them suffer from drug-resistant epilepsy (Kwan et al., 2011; Lin et al., 2014). These individuals might have seizures at any moment, such that their daily lives are influenced by unexpected behavioral changes, loss of muscular control and sudden faint. As a result, a reliable seizure prediction device is becoming an emerging and significant demand to prevent the injury of epileptic coma, or even death. A successful seizure forecast commonly adopts data-driven techniques to monitor the electroencephalography (EEG) signals of the epileptic brain, since such data records rhythmic information induced by coordinated neuronal. The first-in-man study that proves the predictability of seizure has been reported in 2013 (Cook et al., 2013). Since then, many EEG-based studies regarding seizure prediction have been proposed.

### 1.2. Related Work

**Figure 1:**
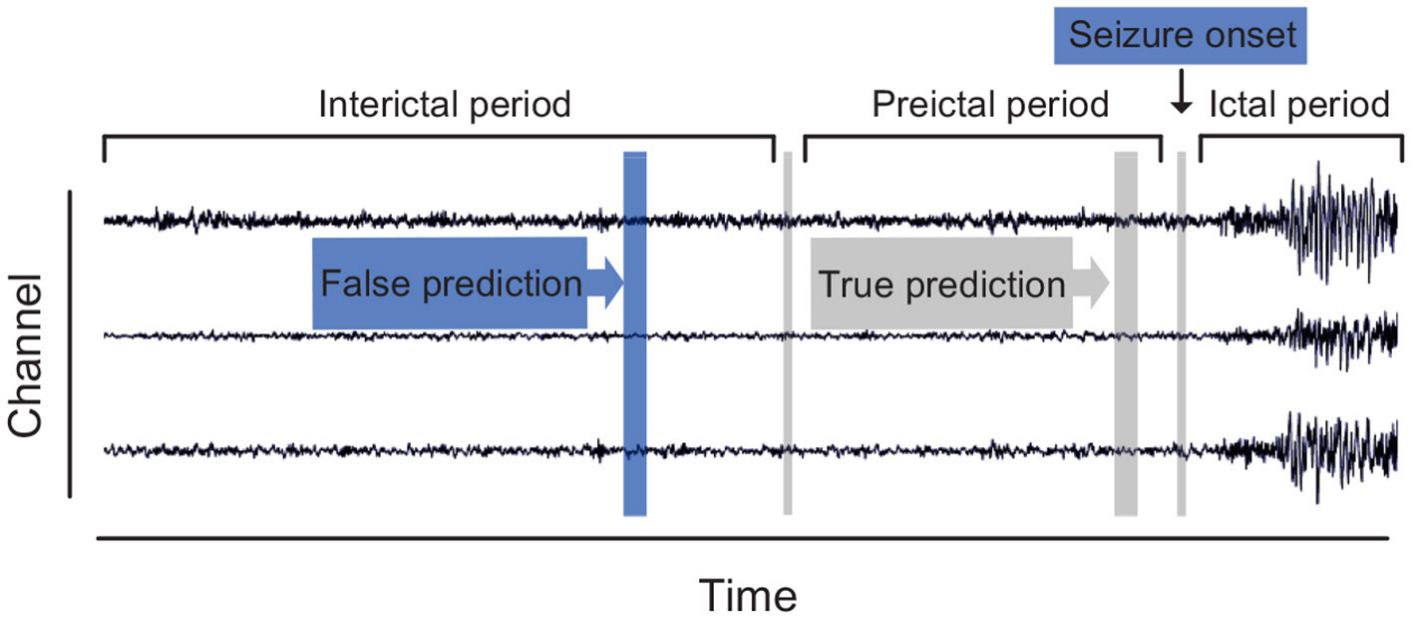
Definition of three brain states in continuous epileptic EEG recordings.

At present, there are two main streams for epileptic seizure prediction. The first stream is a binary classification framework trained to discriminate preictal samples from interictal samples. The ictal and postictal samples are deserted during the training procedure for the uselessness of their contribution to forecast. This stream is widely adopted among researchers in the area of seizure prediction. The second stream assumes that a specific index that fluctuates with changes of seizure stage exists in EEG recordings. These methods attempt to describe this index explicitly and monitor it with a threshold. For instance, spike rate (Li et al., 2013; Karoly et al., 2016; Guo et al., 2017), zero-crossing intervals (Zandi et al., 2013), and phase/amplitude locking value (Myers et al., 2016) have been reported as the indicators. Since a universal preictal biomarker has not been defined explicitly, we also follow the binary scheme of the first stream, which is depicted in Figure 1.

Studies adopting the binary classifier usually combine the features extraction algorithms with machine learning techniques. To be specific, the features extraction algorithms are commonly used in data preprocessing due to the complexity and diversity of EEG signals, and then the machine learning techniques can analyze these features and give their categories. Features extraction approaches like wavelet transform (Vahabi et al., 2015; Moctezuma and Molinas, 2020), Q-factor wavelet transform (Al Ghayab et al., 2019), Fourier neural network (Peng et al., 2021), and fractional Fourier transform (Fei et al., 2017), are employed to learn the high-dimensional representations of samples. Machine learning techniques like support vector machines (Mirowski et al., 2009; Direito et al., 2017; Sun et al., 2019), random forests (Brinkmann et al., 2016), *k*-nearest neighbor (Zhang et al., 2018), and ensemble learning (Peng et al., 2020) are utilized to learn the spatial and temporal representations of seizures. Besides, recently most authors apply deep learning frameworks for seizure prediction. Convolutional neural network (CNN) (Zhang et al., 2019; Lin et al., 2020; Liu et al., 2020), 3D CNN (Ozcan and Erturk, 2019), Long Short-Term Memory (LSTM) Network (Tsiouris et al., 2018; Daoud and Bayoumi, 2019; Li et al., 2020), and cascades of DNN (Özcan and Ertürk, 2017), are introduced to process continuously acquired EEG signals.

### 1.3. Significance

**Figure 2:**
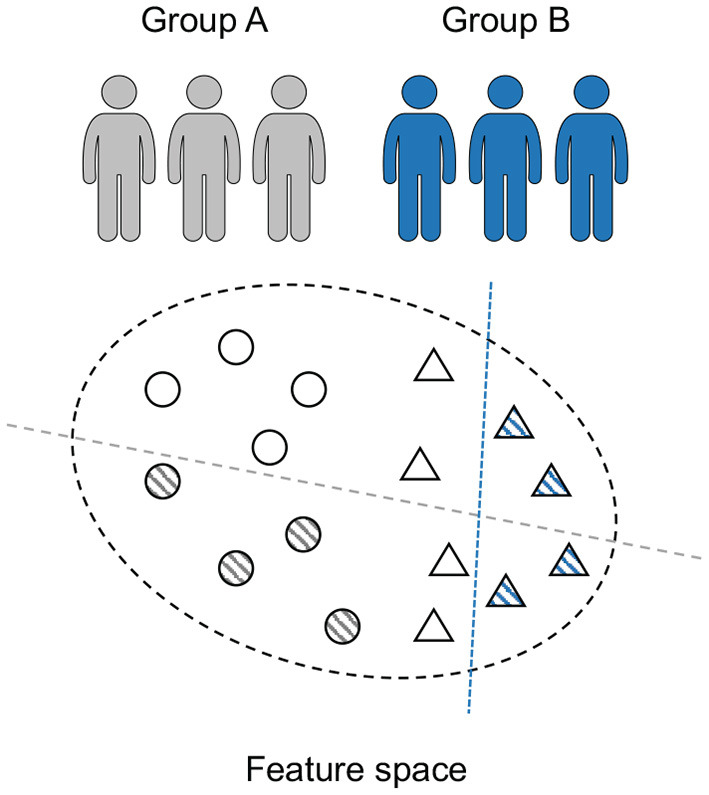
Seizure prediction is a patient-specific problem. The discriminative models (dashed line) of various individuals (circle and triangle) differ significantly.

Although conventional studies report high precision (<85% on average) for the epileptic seizure prediction task, their translation to the practical application is still a challenging issue. The major reason for this situation is that most of these studies only provide patient-specific results. For these patient-specific models, both training and testing sets are recorded from one subject, which leads to limited domain adaptability of previous approaches between different patients (each patient is considered as a domain). As shown in Figure 2, for the patients with epilepsy, the internal patterns vary significantly among various subjects (Jirsa et al., 2017; Elger and Hoppe, 2018; Kuhlmann et al., 2018), which learn totally different discriminative hyperplanes. Therefore, these patient-specific models with good performance might obtain undesired results in real life, although they are significant to personalized medicine. It is obvious that how to develop a predictor that is universal to various patients is the key problem. However, this issue remains unsolved and thus the previous models are not yet in widespread use.

This work attempts to develop a seizure prediction model without the precondition of patient specificity. However, since the underlying patterns and dynamics of epilepsy are not well-understood in neuroscience, the complete desertion of the “target” samples (data of the patient to be tested) is impossible. The training set in existing studies is composed of the “target” data entirely. We attempt to reduce the reliance on the “target” data as much as possible until it reaches a clinically acceptable amount of EEG recordings. To be specific, due to the risk of infection in invasive surgery and the right of privacy, the training set in real life mainly consist of signals of previous patients. And only a small amount of “target” samples can be used for training. We try to simulate this sampling situation to train and test our model.

To achieve a higher generalizationa ability, this study introduces the strategy of domain adaptation (DA) methods for seizure prediction. It is a machine learning technique that can reduce the domain gap. In some successful DA models like maximum mean discrepancy-adversarial autoencoders (MMD-AAE) (Li et al., 2018) and cone manifold domain adaptation (CMDA) (Yair et al., 2019), to minimize the inter-domain distance in a high-dimensional space is the major optimization objective. Inspired by these researches, we hope to develop a generic seizure prediction model based on minimizing the inter-domain distance. To encode the raw EEG data into a high-dimensional space, we design a novel autoencoder using the short-time Fourier transform (STFT) (Cordes et al., 2021) and the MMD-AAE. The main contributions of this study are summarized as follow

- A general seizure prediction model for different patients is proposed based on the MMD-AAE model and STFT technique. It is tested under simulated clinical sampling conditions, making it feasible in practice.
- A domain adaptation framework is developed based on inter-domain distance. This algorithm can improve the generalization ability since it minimizes the domain gap between different patients.
- It is the first study to provide a comparison results of different domain adaptation algorithms on seizure forecast, which is important to follow-up researches.

Based on the MMD-AAE model and STFT technique, the proposed method obtains an above-par generalization ability. Experiments on two open datasets, the Freiburg Hospital EEG database and the CHB-MIT EEG database (Goldberger et al., 2000; Zhou et al., 2018), are conducted for model assessment. Compared with other methods, experimental results indicate that the proposed model achieves high robustness while preserving a decent precision.

## 2. Data Acquisition and Preprocessing

### 2.1. Patients

Two open EEG databases, the Freiburg Hospital intracranial EEG database (Zhou et al., 2018) and the CHB-MIT scalp EEG database (Goldberger et al., 2000), are selected to assess the model performance of our method.

The Freiburg Hospital database includes time series of 87 seizures of 21 subjects with medically intractable focal epilepsy, aged from 10 to 50 years old (8 males and 13 females). EEG signals are recorded invasively by 6 electrodes (3 near the epileptic focus and 3 away from the epileptogenic zone). The sampling rate is 256 Hz for all patients(data of Patient No.12 are sampled at 512 Hz but are down-sampled to 256 Hz).

The CHB-MIT database consists of scalp EEG sequences of 22 epileptic subjects, including 5 males aged from 3 to 22 years and 17 females aged from 1.5 to 19 years. The EEG signals are recorded at a 256 Hz sampling rate using 16-bit analog-to-digital converters. Most samples are collected from 23 channel surface electrodes following the 10-20 standard system for electrode placement (Rojas et al., 2018). Each individual has a subfolder that has 9 to 42 recordings.

### 2.2. Data Selection and Labeling

We use the power line noise removal to denoise the raw EEG recordings. In the intracranial EEG test set, the frequency bands of 47–53 and 97–103 Hz are deserted and in the scalp EEG test set, the frequency bands of 57–63 and 117–123 are discarded. This is because that the noise of the Freiburg database usually occurs at 50 Hz and noise of the CHB-MIT database occurs at 60 Hz. In addition, we perform a patient selection. Only patients who had at least 2 seizures but fewer than 15 per day are chosen, since less than 2 seizures would not be sufficient to support training, and more than 15 would render the prediction meaningless. The subjects chosen in this work are presented in Tables 1, 2.

**Table 1:** Details of the Freiburg Hospital test set.

**Patient**	**Gender**	**Age**	**Seizure type**	**No. of seizures**
Pt 1	F	15	SP	4
Pt 2	M	38	SP, CP, GTC	3
Pt 3	M	14	SP, CP	5
Pt 4	F	26	SP, CP, GTC	5
Pt 5	F	16	SP, CP, GTC	5
Pt 6	F	31	CP, GTC	3
Pt 8	F	32	SP, CP	2
Pt 9	M	44	CP, GTC	4
Pt 10	M	47	SP, CP, GTC	5
Pt 11	F	10	SP, CP, GTC	4
Pt 12	F	42	SP, CP, GTC	3
Pt 13	F	22	SP, CP, GTC	2
Pt 14	F	41	CP, GTC	4
Pt 15	M	31	SP, CP, GTC	4
Pt 16	F	50	SP, CP, GTC	5
Pt 17	M	28	SP, CP, GTC	5
Pt 18	F	25	SP, CP	5
Pt 19	F	28	SP, CP, GTC	4
Pt 20	M	33	SP, CP, GTC	5
Pt 21	M	13	SP, CP	5

*F, Female; M, Male; SP, simple partial; CP, complex partial; GTC, generalized tonic-clonic*.

**Table 2:** Details of the CHB-MIT test set.

**Patient**	**Gender**	**Age**	**Seizure type**	**No. of seizures**
Pt 1	F	11	SP, CP	7
Pt 2	M	11	SP, CP, GTC	3
Pt 3	F	14	SP, CP	6
Pt 5	F	7	CP, GTC	5
Pt 6	F	2	CP, GTC	4
Pt 7	F	15	SP, CP, GTC	3
Pt 8	M	4	SP, CP, GTC	5
Pt 9	F	10	CP, GTC	4
Pt 10	M	3	SP, CP, GTC	6
Pt 13	F	3	SP, CP, GTC	5
Pt 14	F	9	CP, GTC	5
Pt 17	F	12	SP, CP, GTC	3
Pt 18	F	18	SP, CP	6
Pt 19	F	19	SP, CP, GTC	3
Pt 20	F	6	SP, CP, GTC	5
Pt 21	F	13	SP, CP	4

*F, Female; M, Male; SP, simple partial; CP, complex partial; GTC, generalized tonic-clonic*.

The seizure occurrence period (SPO) is set to 0. Only the seizure prediction horizon (SPH) is considered in this study. Thirty minutes before seizure occurrence is set as the SPH. This parameter is given by empirical evidence of comparison experiments applying multiple preictal lengths. If a seizure occurs within 30 min, the forecast model then returns a positive. The raw EEG recordings are split into continuous, non-overlapping segments over a 5-s time window. The sample number for each case is sufficient (> 38,400) to support training. In addition, to obtain equal amounts of preictal and interictal samples, a random subsample on the interictal data is implemented, which circumvents the imbalance of different kinds of training samples.

## 3. Methods

To reduce the impact of inter-individual variability, we propose a generic seizure prediction model. The core idea of our method is to minimize the domain distance between different subjects in the high-dimensional space. Such that domain-invariant features can be extracted during domain distribution alignment. The maximum mean discrepancy (MMD) measure (Zhang et al., 2020) is selected as the distance measure and the high-dimensional space is established by the adversarial autoencoders (AAE) (Makhzani et al., 2015).

### 3.1. Clinical Situation Simulation

The training set of conventional studies is not consistent with the sampling situation in real life. During clinical treatment, it is almost impossible to record a large number of EEG samples from a specific patient over a long period of time. Thus the traditional patient-specific learning strategy can not be performed because the data size is unable to support training. To tackle this issue, we propose a novel predictor that can use other patients' data for training.

**Figure 3:**
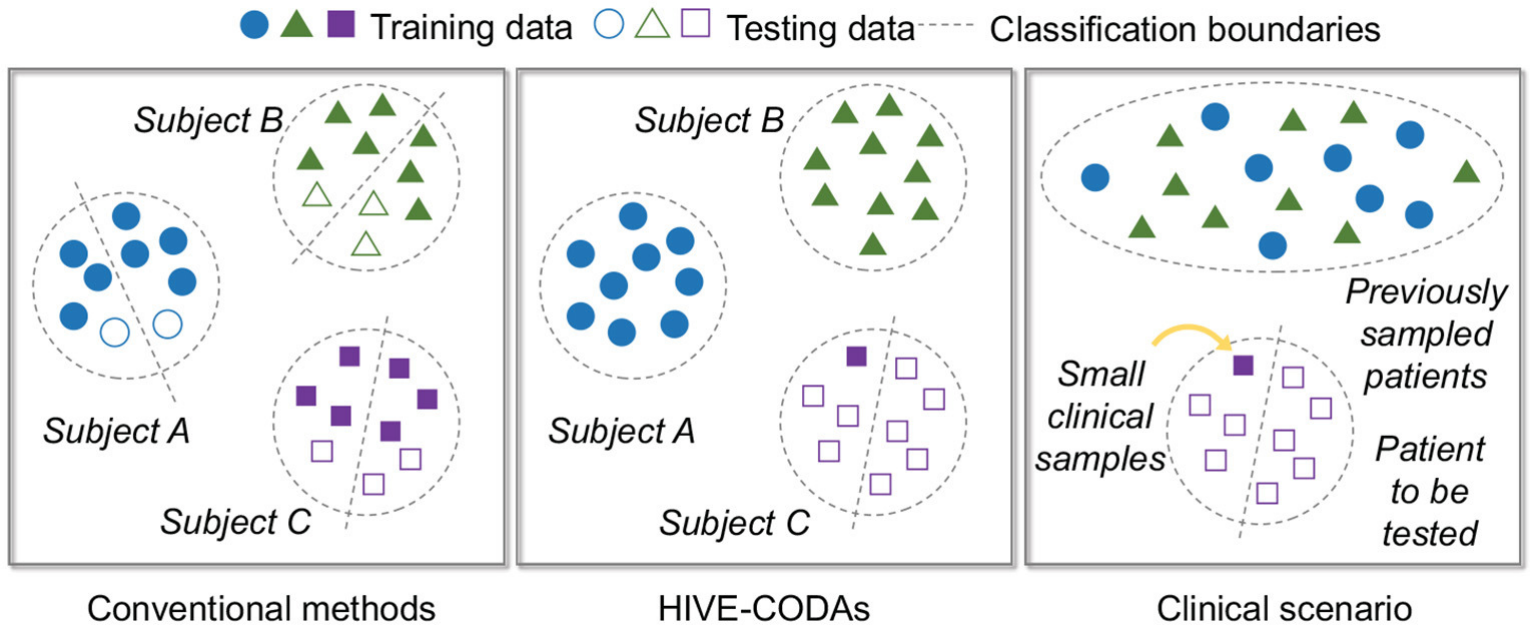
Illustration of clinical situation simulation.

To mimic the sampling situation in the clinic, we adopt a particular training and testing strategy, which is illustrated in Figure 3. To be specific, the training and validation set includes previous patient' data and one seizure from the “target” subject, while the remaining seizures of the “target” subject served as the test set. This strategy refers to the idea of the Leave-one-out cross-validation (LOOCV) approach (Peng et al., 2018). Moreover, the training and validation set are partitioned into 5-folds, and 80% of the data are assigned to the training set while the remaining 20% are assigned to the validation set to prevent overfitting.

### 3.2. Modal Transformation With STFT

Due to the low signal-to-noise ratio (SNR) of EEG recordings, we attempt to transform the input information from time domain into time-frequency domain. Two preprocessing techniques, wavelet and Fourier transforms (Muralidharan et al., 2011; Zhao et al., 2019), are commonly employed to convert EEG segments into image shapes. Here we adopt the short-time Fourier transform (STFT) to produce feature maps from raw EEG sequences. The conversion transforms the EEG time series into matrices, which can meet the input requirement of the two-dimensional MMD-AAE. This procedure can also extract the significant features for seizure prediction.

In the STFT module, the time-varying EEG fragment is converted to a two-dimensional matrix composed of frequency and time axes. Such that an insight in the time-evolution for each time window can be observed by the two-dimensional map. Suppose that there exist *K* domains (patient) in total. The input data from the *K* domains are denoted by $\bar{X}={\left[{\bar{x}}^{\left(1\right)},\cdots \;,{\bar{x}}^{\left(K\right)}\right]}^{T}\in {\mathbb{R}}^{K\times d}$, where $\bar{x}\in {\mathbb{R}}^{d\times 1}$. For an arbitrary domain, the segment with the time index *t* is given as $\bar{x}\left(t\right)$. By performing the STFT procedure, we get the time-frequency feature map of $\bar{x}\left(t\right)$, which is presented as:


(1) $$\begin{array}{l} x\left(\omega ,u\right)=\underset{t}{\sum }\bar{x}\left(t\right)g\left(t-u\right)e^{j\omega t}, \end{array}$$

where ω is the selected frequency band, *g*(*t* − *u*) is the window function. For *K* domains, the STFT is implemented to each subject, and then the inputs are converts to ***X*** = [***x***^(1)^, ⋯ , ***x***^(*K*)^]^*T*^∈ℝ*^K×d^*. The samples of each case are represented by spectrograms. These time-frequency feature maps are then sent to the AAE for invariant feature extraction.

### 3.3. Construction of High-Dimensional Space

**Figure 4:**
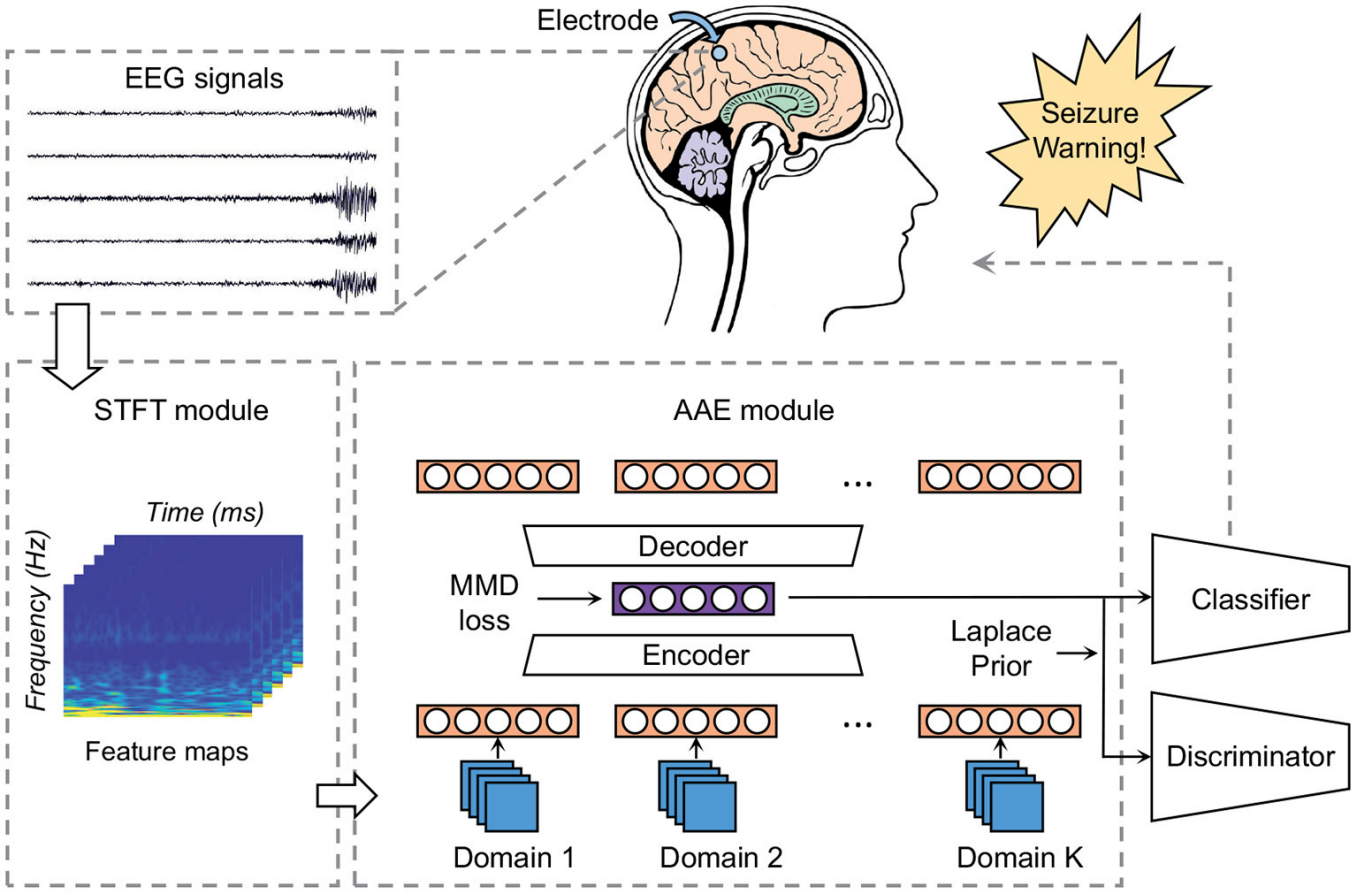
Block diagram of our model: the STFT module converts raw EEG recordings into time-frequency images to meet the input requirement of the AAE module. The AAE module maps each domain's data into a high-dimensional space. MMD loss is employed as the measure to align distributions of different domains. The Laplace prior is exploited to optimize the hidden code *z* using adversarial learning.

This module attempts to establish a high-dimensional space with an encoder and a decoder. The model is illustrated in Figure 4. By using an encoder, we can map the time-frequency images of raw EEG samples into an embedding subspace. And by using a decoder, these hidden layers are then mapped back to a “fake” input matrix. The hidden space is high-dimensional and therefore contains more information. The MMD measure is then utilized to align the distributions of high-dimensional feature vectors between different domains. Thus the optimized hidden code contains sharable information of various patients. We then extract these latent characteristics that are universal among patients for classification.

There are two procedures during the construction of embedding space: the reconstruction process and the distribution alignment process. In the reconstruction process, the autoencoder attempts to recover the time-frequency image from the high-dimensional vectors. The architecture of the autoencoder refers to the structure in MMD-AAE (Li et al., 2018). We set an optimization objective $L_{rec}$ to guide the generated feature map $\tilde{x}$ to match the input map ***x***. The loss function of the reconstruction procedure $L_{rec}$ is defined as:


(2) $$\begin{array}{l} L_{rec}=||\tilde{x}-x{||}_{2}^{2}. \end{array}$$

Now, we specify the form of the inter-domain distance metric. The maximum mean discrepancy (MMD) measure (Jia et al., 2017) is exploited to align the distributions of different domains. Like the reconstruction process, we also give a MMD-based regularization term to optimize the hyperparameters in the neural network (Li et al., 2018). Suppose that ***Z*** = [***z***^(1)^, ⋯ , ***z***^(*K*)^]^*T*^ ∈ ℝ^*K* × *l*^ represents the learned high-dimensional features of *K* domains, where ***z*** ∈ ℝ^*l* × 1^. For two arbitrary hidden vector ***z***^(*i*)^ and ***z***^(*j*)^, we assume that they belong to two unseen probability distribution ℙ^(*i*)^ and ℙ^(*j*)^, respectively. By adopting the kernel embedding technique (Smola et al., 2007), the instance is mapped to a reproducing kernel Hilbert space (RKHS) H. The corresponding mean value in RKHS is given as:


(3) $$\begin{array}{l} \mu \left(\mathbb{P}\right)=E_{z\sim \mathbb{P}}\left[h\left(z,\cdot \right)\right], \end{array}$$

where is μ(·) the mean map operation. *h*(***z***, ·) is the kernel function induced by the feature map in H. In this work, we adopt the RBF kernel following the MMD-AAE model (Li et al., 2018). The inter-domain distance between the latent codes ***z***^(*i*)^ and ***z***^(*j*)^ can be described as:


(4) $$\begin{array}{l} dis\left(z^{\left(i\right)},z^{\left(j\right)}\right)=||\mu_{{\mathbb{P}}^{\left(i\right)}}-\mu_{{\mathbb{P}}^{\left(j\right)}}|{|}_{H}. \end{array}$$

Then, it is obvious that the regularization item of the entire latent space can be defined as:


(5) $$\begin{array}{l} R_{dis}\left(z^{\left(1\right)},\cdots \;,z^{\left(K\right)}\right)=\frac{1}{K^{2}}\underset{1\leq i,j\leq K}{\sum }dis\left(z^{\left(i\right)},z^{\left(j\right)}\right). \end{array}$$

With the distance error above, the extracted high-dimensional features can generalize well across all the domains, since the neural network learns their common code by aligning their distributions.

### 3.4. Optimization Using Adversarial Learning

To further optimize the learned features in section 3.3, we introduce an adversarial learning-based module according to AAE (Makhzani et al., 2015). Adversarial learning is an emerging machine learning approach in recent years, which has been successfully applied in the area of epileptic EEG signal processing. It usually contains a generator network *G* with parameters Θ_*G*_ and a discriminator network *D* with parameters Θ_*D*_. The generator network *G* will produce some fake version of the inputs. These fake data are sent to the discriminator network *D* together with the real input data. Then the discriminator network *D* will tell whether the input sample is artificially generated. During the training procedure, the neural network finds a Nash equilibrium between the generator and the discriminator, and the fake data are gradually approaching the real one. This “zero-sum game” can be described as:


(6) $$\underset{G}{\min}\underset{D}{\;\max}\;E_{x_{r}\sim p_{r}}\left[\log D_{\Theta_{D}}\left(x_{r}\right)\right]-E_{x_{f}\sim p_{f}}\left[\log D_{\Theta_{D}}\left(x_{f}\right)\right],$$

where ***x***_*r*_ and *p*_*r*_ are the real data and the corresponding distribution, ***x***_*f*_ and *p*_*f*_ are the fake version. After the optimization with the loss $J_{gan}$, the adversarial subnetwork can align *p*_*f*_ to the constant prior *p*_*r*_.

We hope to utilize the aforementioned principle in the embedding space. Therefore, we assume that the “true” universal features among different patients comes from an arbitrary prior distribution *p*(***z***). The adversarial module draws samples from the prior distribution *p*(***z***) and considers these samples as the real data ***z***_*r*_. Accordingly, the learned latent information ***z*** is considered as the fake data ***z***_*f*_, where the autoencoder represents the generator *G*. A discriminator *D* is also implemented in the adversarial module, which distinguishes the produced vector ***z*** from the samples of the prior. In this study, the prior distribution is selected as the Laplace distribution ***z*** ~ *Laplace*(η), where η denotes the hyperparameter.

The training strategy of the adversarial module is a variational inference process. To be specific, first, the latent coding space has been established by the encoder explicitly. Then the distributions among different domains are aligned with the MMD regularization item to extract the domain-invariant feature vectors. These features are guided to approach a prior distribution *p*(***z***). To match the hidden code with an arbitrary distribution can effectively alleviate the overfitting to a certain patient. After the optimization process, the aggregated posterior distribution *q*(***z***) of the hidden layer is as follows:


(7) $$\begin{array}{l} q\left(z\right)=\underset{x}{\int }q\left(z|x\right)p_{d}\left(x\right)dx, \end{array}$$

where *q*(***z***∣***x***) is the encoding function of the autoencoder, *p*_*d*_(***x***) is the marginal distribution of data. During the training phase, the probabilistic autoencoder is regularized with an adversarial loss function $J_{adv}$, which is described as:


(8) $$\begin{array}{l} \begin{array}{c} J_{adv}=E_{z\sim p\left(z\right)}\left[\log D\left(z\right)\right]\\ \;+E_{x\sim p_{d}}\left[\log\left(1-D\left(G\left(x\right)\right)\right)\right]. \end{array} \end{array}$$

After training, a generative model is defined by imposing the prior *p*(***z***) on the data distribution. A one-hot encoding vector ***y*** is used for supervised learning (Kumar et al., 2018; Saito et al., 2020). Then we use the learned domain-invariant features for seizure prediction. An SVM classifier is introduced to analyze the extracted features. The loss function of the classification procedure is denoted by $L_{cla}$. The objective function of the entire model can be defined as:


(9) $$\underset{G,C}{\min}\underset{D}{\;\max}\;L_{cla}+\lambda_{0}L_{rec}+\lambda_{1}J_{adv}+\lambda_{2}R_{dis},$$

where λ_0_, λ_1_ and λ_2_ represent the trade-off positive parameters, and *C* is the classifier. Our model is optimized jointly with these modules. In general, the MMD-based regularization term is designed to align the distributions among different patients. The AAE architecture is used to construct the latent feature space. The adversarial module is developed to match the hidden code with a prior distribution. Thus this model can circumvent the overfitting to a certain patient.

## 4. Results and Discussion

In this section, comparison experiments are conducted to verify the generalization ability and evaluate the forecast precision. Our model is tested on both intracranial and scalp EEG signals. Three commonly-used indicators about model performance are exploited in experiments: sensitivity, false alarm rate per hour (FPR), and area under the receiver operating characteristic curve (AUC). Noted that each EEG fragment represents an event so that the event-based indicators are used for evaluation (Temko et al., 2011).

### 4.1. Comparison With Conventional Methods

**Figure 5:**
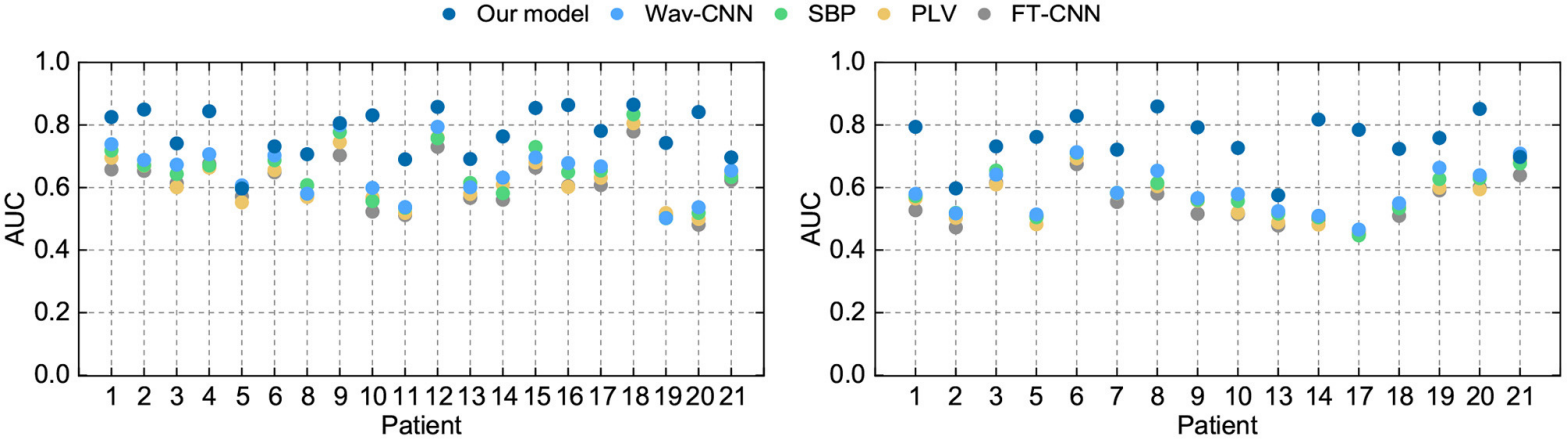
AUC of different seizure prediction models on the Freiburg Hospital test set **(left)** and the CHB-MIT test set **(right)**.

To demonstrate the advantages over conventional methods, we select four seizure prediction researches for comparison: FT-CNN (Truong et al., 2018), phase locking value (PLV) (Cho et al., 2016), spectral band power (SBP) (Ozcan and Erturk, 2019), and Wav-CNN (Khan et al., 2017). All these approaches have obtained good model performance when the training and testing processes are performed on the same subject. But data from previous cases are not used in their training phases. Here we train these models with EEG samples from multiple patients and test them with the “unseen” patient's data. The sensitivity and FPR are provided in Tables 3, 4. The AUC value for each patient is illustrated in Figure 5.

**Table 3:** Results compared with conventional methods on the Freiburg Hospital database.

**Source**	**Target**	**FT-CNN**		**PLV**		**SBP**		**Wav-CNN**		**Our model**	
		***S*_n_**	**FPR (/h)**	***S*_n_**	**FPR (/h)**	***S*_*n*_**	**FPR (/h)**	***S*_*n*_**	**FPR (/h)**	***S*_*n*_**	**FPR (/h)**
S.C.	Pt 1	0.64	0.21	0.66	0.20	0.69	0.19	0.70	0.17	0.79	0.16
S.C.	Pt 2	0.63	0.3	0.65	0.28	0.66	0.26	0.67	0.24	0.82	0.12
S.C.	Pt 3	0.58	0.24	0.59	0.23	0.62	0.22	0.64	0.22	0.74	0.20
S.C.	Pt 4	0.64	0.25	0.65	0.24	0.66	0.22	0.69	0.20	0.83	0.16
S.C.	Pt 5	0.56	0.4	0.58	0.39	0.59	0.38	0.60	0.38	0.57	0.30
S.C.	Pt 6	0.64	0.27	0.64	0.26	0.67	0.26	0.69	0.26	0.73	0.18
S.C.^*^	Pt 8	0.54	0.33	0.55	0.33	0.57	0.32	0.57	0.31	0.68	0.29
S.C.	Pt 9	0.70	0.18	0.72	0.17	0.75	0.15	0.77	0.13	0.77	0.19
S.C.	Pt 10	0.52	0.34	0.53	0.33	0.55	0.32	0.58	0.30	0.81	0.16
S.C.	Pt 11	0.50	0.32	0.5	0.30	0.52	0.29	0.52	0.28	0.68	0.29
S.C.	Pt 12	0.72	0.15	0.74	0.13	0.75	0.12	0.77	0.13	0.82	0.09
S.C.^*^	Pt 13	0.55	0.27	0.56	0.25	0.59	0.24	0.60	0.23	0.66	0.29
S.C.	Pt 14	0.56	0.46	0.57	0.46	0.58	0.44	0.60	0.43	0.75	0.22
S.C.	Pt 15	0.66	0.17	0.66	0.16	0.69	0.15	0.69	0.13	0.83	0.12
S.C.	Pt 16	0.59	0.33	0.6	0.32	0.63	0.31	0.65	0.30	0.85	0.12
S.C.	Pt 17	0.59	0.34	0.62	0.33	0.63	0.31	0.65	0.30	0.77	0.21
S.C.	Pt 18	0.76	0.14	0.78	0.13	0.80	0.11	0.83	0.12	0.84	0.09
S.C.	Pt 19	0.48	0.29	0.48	0.28	0.48	0.27	0.5	0.26	0.73	0.23
S.C.	Pt 20	0.45	0.33	0.47	0.33	0.48	0.33	0.51	0.32	0.82	0.15
S.C.	Pt 21	0.60	0.28	0.62	0.27	0.62	0.25	0.65	0.24	0.66	0.31
Avg.		0.59	0.28	0.61	0.27	0.63	0.26	0.64	0.25	0.76	0.19

*S.C., simulated clinical samples; S_n_, sensitivity; FPR, false prediction rate; Avg., average result. S.C.*

* *uses NO samples of the predictor user*.

**Table 4:** Results compared with conventional methods on the CHB-MIT database.

**Source**	**Target**	**FT-CNN**		**PLV**		**SBP**		**Wav-CNN**		**Our model**	
		***S*_*n*_**	**FPR (/h)**	***S*_*n*_**	**FPR (/h)**	***S*_*n*_**	**FPR (/h)**	***S*_*n*_**	**FPR (/h)**	***S*_*n*_**	**FPR (/h)**
S.C.	Pt 1	0.52	0.33	0.54	0.31	0.55	0.31	0.56	0.31	0.77	0.25
S.C.	Pt 2	0.46	0.37	0.47	0.37	0.48	0.34	0.49	0.32	0.56	0.32
S.C.	Pt 3	0.59	0.30	0.60	0.30	0.63	0.29	0.63	0.28	0.70	0.24
S.C.	Pt 5	0.48	0.39	0.48	0.37	0.49	0.35	0.51	0.34	0.74	0.23
S.C.	Pt 6	0.64	0.3	0.66	0.29	0.68	0.28	0.70	0.28	0.79	0.27
S.C.	Pt 7	0.53	0.21	0.56	0.21	0.56	0.29	0.57	0.26	0.71	0.15
S.C.	Pt 8	0.58	0.26	0.60	0.25	0.61	0.24	0.63	0.23	0.82	0.22
S.C.	Pt 9	0.51	0.34	0.54	0.33	0.55	0.33	0.56	0.32	0.78	0.20
S.C.	Pt 10	0.5	0.31	0.51	0.29	0.53	0.28	0.54	0.26	0.72	0.24
S.C.	Pt 13	0.46	0.21	0.47	0.20	0.50	0.28	0.50	0.27	0.54	0.37
S.C.	Pt 14	0.46	0.38	0.48	0.38	0.49	0.36	0.50	0.34	0.80	0.14
S.C.	Pt 17	0.42	0.37	0.43	0.35	0.44	0.35	0.44	0.35	0.75	0.3
S.C.	Pt 18	0.49	0.29	0.52	0.29	0.53	0.27	0.54	0.25	0.70	0.22
S.C.	Pt 19	0.56	0.28	0.58	0.27	0.60	0.25	0.63	0.23	0.73	0.19
S.C.	Pt 20	0.57	0.24	0.59	0.22	0.60	0.2	0.62	0.28	0.82	0.16
S.C.	Pt 21	0.63	0.25	0.66	0.24	0.67	0.22	0.70	0.20	0.68	0.28
Avg.		0.51	0.30	0.54	0.29	0.56	0.29	0.57	0.28	0.73	0.24

*S.C., simulated clinical samples; S_n_, sensitivity; FPR, false prediction rate; Avg., average result*.

The widely-used Freiburg Hospital database is employed in this work to evaluate our model on the intracranial EEG. Table 3 illustrates that our model outperforms all the other conventional methods in a clear margin. It is reasonable since the prior studies adopt the patient-specific strategy and consider little about the domain adaptability. Conversely, our method exhibits obvious advantages in terms of generalization ability, which achieves a sensitivity of 76% and an FPR of 0.19/h on average. Although these results do not achieve the high accuracy of tests under the patient-specific conditions, such precision can still meet the daily needs of patients with epilepsy because they are similar to the warning frequency in the first-in-man trial (Cook et al., 2013).

Still, the simulated clinical sampling situation is “harsh” for the forecast task. It can be observed that the performances of all these models are not desired. Moreover, on several outliers like Pt 5, 11, and 21, the performance degradation is particularly noticeable. It might be caused by a more complex internal mode in the high-dimensional space. Note that even on these outliers, the sensitivity of our model is slightly higher than other methods, which demonstrates that our approach achieves better robustness.

As for the scalp EEG recordings, we test these methods using the public CHB-MIT database, produced by the Massachusetts Institute of Technology. As shown in Table 4, our algorithm achieves a sensitivity of 73% and an FPR of 0.24/h on average. The results of our model show a significant improvement compared with the conventional methods, which is consistent with the results on intracranial EEG. However, all the model performances drop to a varying degree compared with the precisions on the Freiburg test set. It might be caused by the low spatial resolution of the scalp EEG signals for they are more susceptible to being contaminated by noises (Ramantani et al., 2016; Usman et al., 2019). In other words, intracranial EEG recordings have the higher SNR and the artifacts are typically seen in scalp EEG.

There are also some outliers in the tests on the scalp EEG signal. On Pt 2, 13, and 21, all these models obtain a subpar performance. Larger domain gaps might exist in the sample space of these outliers, which makes the hyperplane difficult to capture. Even on these outliers, the precision of our model is slightly higher than the lower bound of a random binary classifier. It gives us confidence in applying DA techniques to epileptic seizure prediction.

### 4.2. Comparison With DA Methods

**Figure 6:**
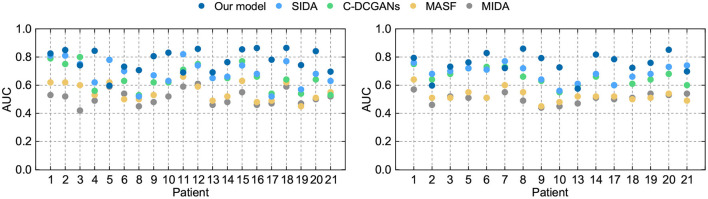
AUC of different DA models on the Freiburg Hospital test set **(left)** and the CHB-MIT test set **(right)**.

We further compare our model with domain adaptation (DA) methods in the existing literature. However, few applications regarding DA approaches have been reported in the area of epileptic seizure prediction. Thus we have to employ DA methods from other fields. The maximum independence domain adaptation (MIDA) (Yan et al., 2017), model-agnostic learning of semantic features (MASF), conditional deep convolutional generative adversarial networks (C-DCGANs) (Zhang et al., 2021) and subject-invariant domain adaption (SIDA) (Rayatdoost et al., 2021) are introduced to verify the advantage of our model. The sensitivity and FPR are provided in Tables 5, 6. The AUC value for each patient is illustrated in Figure 6.

**Table 5:** Results compared with DA methods on the Freiburg Hospital database.

**Source**	**Target**	**MIDA**		**MASF**		**C-DCGANs**		**SIDA**		**Our model**	
		***S*_*n*_**	**FPR (/h)**	***S*_*n*_**	**FPR (/h)**	***S*_*n*_**	**FPR (/h)**	***S*_*n*_**	**FPR (/h)**	***S*_*n*_**	**FPR (/h)**
S.C.	Pt 1	0.57	0.22	0.62	0.21	0.78	0.19	0.83	0.18	0.79	0.16
S.C.	Pt 2	0.56	0.28	0.60	0.26	0.73	0.26	0.82	0.25	0.82	0.12
S.C.	Pt 3	0.52	0.23	0.62	0.22	0.77	0.22	0.78	0.20	0.74	0.2
S.C.	Pt 4	0.49	0.23	0.60	0.23	0.61	0.21	0.62	0.20	0.83	0.16
S.C.	Pt 5	0.57	0.37	0.53	0.35	0.78	0.35	0.79	0.33	0.57	0.30
S.C.	Pt 6	0.53	0.23	0.60	0.23	0.63	0.23	0.70	0.23	0.73	0.18
S.C.^*^	Pt 8	0.45	0.33	0.51	0.33	0.53	0.33	0.55	0.30	0.68	0.29
S.C.	Pt 9	0.49	0.37	0.51	0.36	0.68	0.26	0.70	0.24	0.77	0.19
S.C.	Pt 10	0.52	0.33	0.54	0.32	0.62	0.32	0.64	0.31	0.81	0.16
S.C.	Pt 11	0.59	0.33	0.57	0.32	0.67	0.30	0.79	0.31	0.68	0.29
S.C.	Pt 12	0.59	0.36	0.63	0.34	0.73	0.24	0.75	0.22	0.82	0.09
S.C.^*^	Pt 13	0.45	0.29	0.56	0.29	0.69	0.27	0.69	0.26	0.66	0.29
S.C.	Pt 14	0.45	0.46	0.52	0.45	0.65	0.44	0.60	0.44	0.75	0.22
S.C.	Pt 15	0.56	0.16	0.67	0.16	0.52	0.36	0.74	0.16	0.83	0.12
S.C.	Pt 16	0.44	0.35	0.48	0.33	0.76	0.33	0.64	0.31	0.85	0.12
S.C.	Pt 17	0.44	0.36	0.47	0.35	0.53	0.32	0.52	0.32	0.77	0.21
S.C.	Pt 18	0.58	0.36	0.61	0.35	0.77	0.22	0.77	0.21	0.84	0.09
S.C.	Pt 19	0.45	0.29	0.47	0.28	0.53	0.27	0.58	0.26	0.73	0.23
S.C.	Pt 20	0.47	0.34	0.53	0.34	0.62	0.31	0.66	0.31	0.82	0.15
S.C.	Pt 21	0.52	0.30	0.54	0.29	0.52	0.27	0.66	0.26	0.66	0.31
Avg.		0.51	0.31	0.56	0.30	0.66	0.29	0.69	0.27	0.76	0.19

S.C., simulated clinical samples; S_n_, sensitivity; FPR, false prediction rate; Avg., average result. S.C.^*^ uses NO samples of the predictor user.

**Table 6:** Results compared with DA methods on the CHB-MIT database.

**Source**	**Target**	**MIDA**		**MASF**		**C-DCGANs**		**SIDA**		**Our model**	
		***S*_*n*_**	**FPR (/h)**	***S*_*n*_**	**FPR (/h)**	***S*_*n*_**	**FPR (/h)**	***S*_*n*_**	**FPR (/h)**	***S*_*n*_**	**FPR (/h)**
S.C.	Pt 1	0.55	0.34	0.61	0.33	0.74	0.30	0.74	0.28	0.77	0.25
S.C.	Pt 2	0.43	0.38	0.49	0.38	0.66	0.37	0.64	0.35	0.56	0.32
S.C.	Pt 3	0.51	0.28	0.50	0.27	0.65	0.25	0.67	0.24	0.70	0.24
S.C.	Pt 5	0.48	0.42	0.51	0.40	0.69	0.37	0.69	0.36	0.74	0.23
S.C.	Pt 6	0.46	0.29	0.52	0.27	0.72	0.27	0.72	0.25	0.79	0.27
S.C.	Pt 7	0.54	0.25	0.56	0.24	0.73	0.21	0.73	0.19	0.71	0.15
S.C.	Pt 8	0.48	0.27	0.60	0.26	0.67	0.25	0.66	0.24	0.82	0.22
S.C.	Pt 9	0.46	0.31	0.51	0.29	0.58	0.27	0.57	0.25	0.78	0.20
S.C.	Pt 10	0.45	0.28	0.46	0.27	0.52	0.25	0.52	0.24	0.72	0.24
S.C.	Pt 13	0.48	0.21	0.51	0.39	0.61	0.38	0.62	0.26	0.54	0.37
S.C.	Pt 14	0.47	0.39	0.48	0.39	0.64	0.36	0.65	0.35	0.80	0.14
S.C.	Pt 17	0.49	0.38	0.50	0.37	0.61	0.37	0.59	0.35	0.75	0.30
S.C.	Pt 18	0.50	0.30	0.49	0.30	0.57	0.28	0.61	0.28	0.70	0.22
S.C.	Pt 19	0.51	0.39	0.51	0.36	0.62	0.34	0.62	0.24	0.73	0.19
S.C.	Pt 20	0.53	0.25	0.55	0.23	0.70	0.23	0.66	0.21	0.82	0.16
S.C.	Pt 21	0.50	0.27	0.52	0.26	0.63	0.24	0.68	0.22	0.68	0.28
Avg.		0.49	0.31	0.52	0.31	0.65	0.30	0.65	0.27	0.73	0.24

*S.C., simulated clinical samples*.

For the intracranial EEG samples, we still exploit the Freiburg Hospital database as the test set. Evidently, compared with other DA methods, our model achieves the best performance with a sensitivity of 76% and an FPR of 0.19/h on average. Then the SIDA method exhibits a slight advantage over other methods. Comparing this model to SIDA, we see a benefit of approximately 7% is obtained. Comparing our method to C-DCGANs, we remark that a further 3% benefit is obtained. As for the MASF, a benefit of 20% is observed, for a total of 25% margin over MIDA.

In terms of the scalp EEG data, the open CHB-MIT dataset is applied in the experiment. The order of precision of these DA algorithms is consistent with the performance of the intracranial EEG data. Our model has obvious advantages over other approaches with a sensitivity of 73% and an FPR of 0.24/h on average. The high model performance of our model on both intracranial and scalp EEG testifies to the application potential on seizure forecast.

Evidently, SIDA achieves the best performance except for the proposed method. The reason for this superiority might credit the combination of CNN and generative adversarial network (GAN). SIDA is the deep neural network from the field of emotion recognition. Raw EEG data are converted to spectrum in EEG. By minimizing loss of emotion recognition and subject confusion, SIDA extracts the invariant features among different domains. We conjecture that the architecture of GAN in SIDA might have advantages in this task, which needs to be further proved.

The C-DCGANs makes a relatively larger contribution compared with other modules. An adversarial learning-based structure is also employed by C-DCGANs. It also uses the data augmentation technique, which generates EEG recordings artificially. By increasing the data diversity, C-DCGANs hopes to improve the domain shift robustness. Nevertheless, the man-made data may involve more artifacts (Fahimi et al., 2020) that contaminate EEG samples. Besides, C-DCGANs is a variation of deep learning-based frameworks. As such, it has uncertainties associated with DNN, in particular a lack of formal convergence guarantees.

Not surprisingly, the results of MASF and MIDA are not satisfactory. The core idea of MASF is to establish a model-agnostic learning paradigm using semantic features and gradient-based meta-learning. However, the discriminant hyperplane in the high-dimensional space might be too complex to be described with semantic features. MIDA reduces differences between distributions of domains by learning a subspace with background information. It is obvious that the background-specific features are not valid characteristics.

Based on the aforementioned observations, we conjecture that adversarial learning-based techniques are relatively superior for alleviating individual variability, since all the adversarial learning-based models achieve a decent model performance and generalization ability for seizure prediction. Experiments on both intracranial and scalp EEG datasets suggest that adversarial structure has potential in developing a generic seizure forecast model.

### 4.3. Impact on Different Components

In this section, we conduct experiments to understand the impact of different modules of the proposed model on the final forecast results. To calculate the contribution of each component quantitatively, we adjust the corresponding trade-off positive parameters and observe the variation tendency. The experiment results are listed in Tables 7, 8. Here we discuss three components in this model: the reconstruction module with a loss $L_{rec}$, the adversarial module with a loss $J_{adv}$, and the inter-domain distance regularization term $R_{dis}$.

**Table 7:** Comparison results on the Freiburg Hospital database using various components.

**Method**	***S*_*n*_**	**FPR (/h)**	**Acc**	**AUC**
No $R_{dis}$	0.60 ± 0.03	0.35 ± 0.03	0.63 ± 0.04	0.63 ± 0.03
No $J_{adv}$	0.67 ± 0.03	0.31 ± 0.03	0.68 ± 0.03	0.69 ± 0.03
No $L_{rec}$	0.71 ± 0.03	0.27 ± 0.03	0.72 ± 0.03	0.72 ± 0.03
Our model	0.76 ± 0.03	0.19 ± 0.03	0.78 ± 0.03	0.78 ± 0.03

*S_n_, sensitivity; FPR, false positive rate; Acc, accuracy*.

**Table 8:** Comparison results on the CHB-MIT database using various components.

**Method**	***S_n_***	**FPR (/h)**	**Acc**	**AUC**
No $R_{dis}$	0.57 ± 0.03	0.36 ± 0.03	0.59 ± 0.03	0.59 ± 0.03
No $J_{adv}$	0.64 ± 0.04	0.33 ± 0.03	0.66 ± 0.04	0.66 ± 0.03
No $L_{rec}$	0.68 ± 0.03	0.29 ± 0.03	0.69 ± 0.03	0.70 ± 0.03
Our model	0.73 ± 0.03	0.24 ± 0.03	0.75 ± 0.03	0.75 ± 0.03

*S_n_, sensitivity; FPR, false positive rate; Acc, accuracy*.

As shown in Tables 7, 8, we observe that removing the inter-domain distance regularization item, the adversarial subnetwork, or the classification component causes performance drop on both intracranial and scalp EEG databases. Such results indicate that these modules can effectively improve the model performance: (1) AAE is suitable for epileptic EEG signal processing, and the embedding space made by AAE is meaningful. (2) MMD is an appropriate distance measure to minimize the domain gaps in the seizure forecast task. (3) The reconstruction procedure can force the model to learn the significant features from the latent high-dimensional space.

By adjusting these trade-off items, a set of hyperparameters that are suitable for seizure prediction can be obtained. For the intracranial EEG data, the most appropriate trade-off parameters are set as λ_0_ = 1.05, λ_1_ = 1.2*e*2, λ_2_ = 0.7. For the scalp EEG data, the most appropriate parameter are set as λ_0_ = 1, λ_1_ = 1.1*e*2, λ_2_ = 0.6.

**Figure 7:**
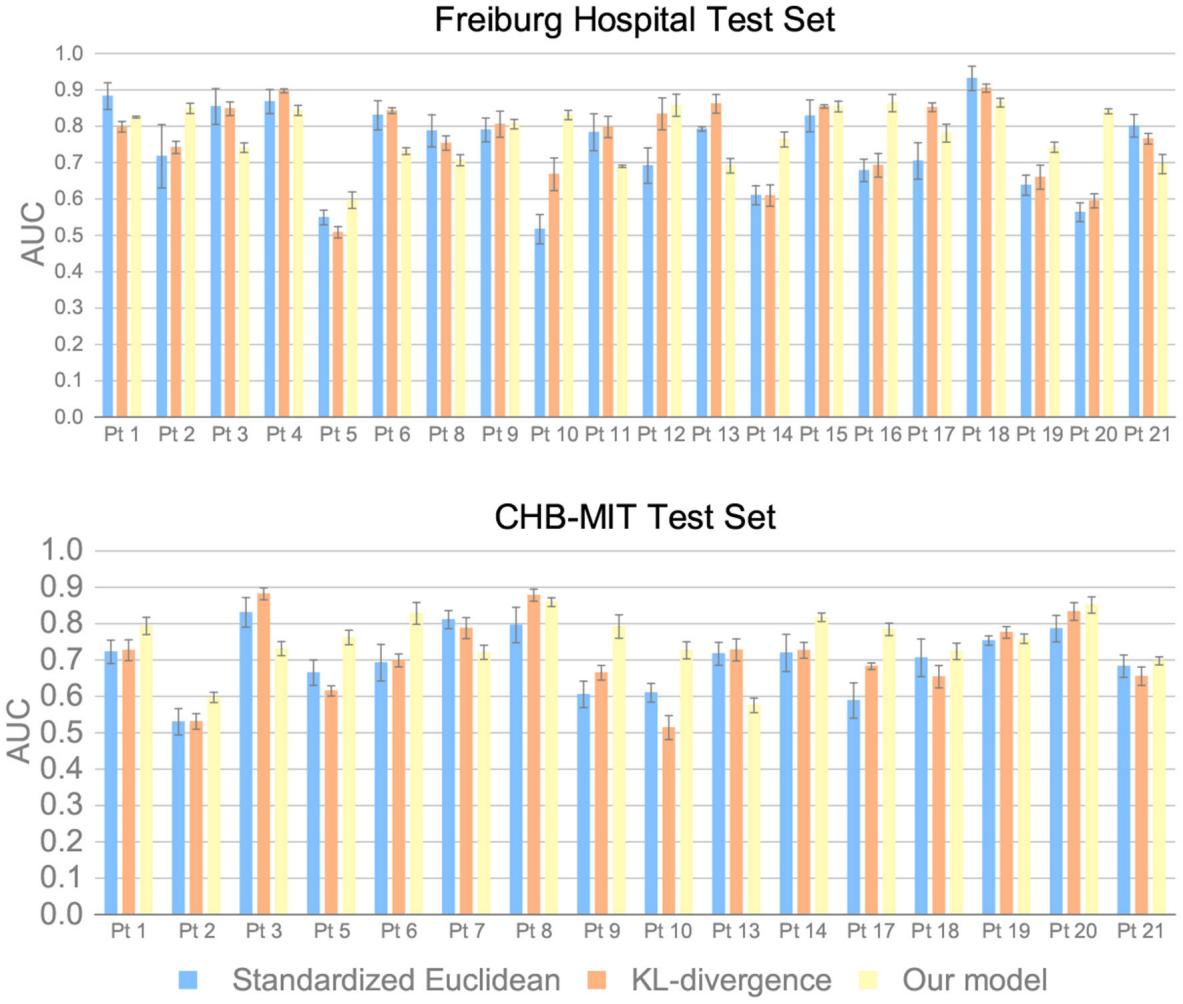
AUC of different inter-domain distance measures on the Freiburg Hospital test set and the CHB-MIT test set.

We also discuss the superiority of the MMD measure over other distance metrics. The standardized Euclidean distance and the KL-divergence are used for comparison. Experimental results are provided in Figure 7. The results suggest that the precision can increase by 3% for intracranial EEG and 4% for scalp EEG by applying the MMD measure, which demonstrates the advantage of MMD measure on the seizure prediction task.

## 5. Conclusion

This work proposes a generic seizure predictor to alleviate the impact of individual variability. By combining STFT with MMD-AAE, our model reduces the effects of epileptic domain variance and improves the generalization ability. Besides, a simulated clinical sampling scenario is used during training and testing periods, which is the first attempt to adopt this assessing strategy. Compared with the patient-specific strategy from previous researches, such a test approach is relatively challenging. Nonetheless, our method achieves high domain shift robustness and precision, which demonstrates its feasibility of real-world applications.

By analyzing the comparison results of DA methods, a conjecture about the effectiveness of adversarial learning in epileptic seizure prediction is obtained. The underlying causes of this phenomenon remain unclear because there is no definitive explanation of the dynamics of epilepsy in the existing literature. The search for more powerful DA algorithms and the underlying reasons will be considered as part of our future research.

## Data Availability Statement

The datasets presented in this study can be found in online repositories. The names of the repository/repositories and accession number(s) can be found in the article/supplementary material.

## Ethics Statement

Ethical review and approval were not required for the current study in accordance with the local legislation and institutional requirements. Written informed consent was obtained from the individual(s), and minor(s)' legal guardian/next of kin, for the publication of any potentially identifiable images or data included in this article.

## Author Contributions

PP, LY, and HW designed the experiments. PP and HW analyzed the experimental results. All authors carried out the experiments, wrote the manuscript, contributed to the article, and approved the submitted version.

## Funding

This work was supported in part by the National Natural Science Foundation of China (Grant Nos. 61802059 and 61773118), in part by the National Key R&D Program of China (Grant No. 2018YFB1500800), in part by the Science and Technology Project of State Grid Corporation of China, Intelligent operation and maintenance technology of distributed photovoltaic system (Grant No. SGTJDK00DYJS2000148), in part by the Natural Science Foundation of Jiangsu (Grant No. BK20180365), in part by Zhishan Young Scholar Program of Southeast University and the Fundamental Research Funds for the Central Universities (Grant No. 2242021R41118).

## Conflict of Interest

The authors declare that the research was conducted in the absence of any commercial or financial relationships that could be construed as a potential conflict of interest.

## Publisher's Note

All claims expressed in this article are solely those of the authors and do not necessarily represent those of their affiliated organizations, or those of the publisher, the editors and the reviewers. Any product that may be evaluated in this article, or claim that may be made by its manufacturer, is not guaranteed or endorsed by the publisher.
